# A High-Throughput Antiviral Assay Based on a Sindbis Virus-GFP for the Discovery of Inhibitors of Alphavirus Replication

**DOI:** 10.3390/v18030290

**Published:** 2026-02-27

**Authors:** Gabriel Augusto Pires de Souza, Rana Abdelnabi, Bert Vanmechelen, Leni Van Eyck, Nelleke Cloet, Deniz Öner, Dirk Roymans, Aleksandra Siekierska, Koert Stittelaar, Johan Neyts

**Affiliations:** 1 VirusBank Platform, Department of Microbiology, Immunology and Transplantation, Rega Institute, KU Leuven, 3001 Leuven, Belgiumrana.abdelnabi@kuleuven.be (R.A.); bert.vanmechelen@kuleuven.be (B.V.); leni.vaneyck@kuleuven.be (L.V.E.); nelleke.cloet@cistim.be (N.C.); ola.siekierska@kuleuven.be (A.S.); koert.stittelaar@kuleuven.be (K.S.); 2 Virology, Antiviral Drug & Vaccine Research Group, Department of Microbiology, Immunology and Transplantation, Rega Institute, KU Leuven, 3000 Leuven, Belgium; 3 Centre for Drug Design and Discovery (CD3), Bio-Incubator 2, Gaston Geenslaan 2, 3001 Leuven, Belgium

**Keywords:** emerging viruses, alphavirus, Sindbis virus, chikungunya virus, antiviral screening, high-throughput screening

## Abstract

The re-emergence of alphaviruses (family *Togaviridae*), such as chikungunya virus, poses significant public health risks, with direct impact on quality of life and work productivity. There are no approved antiviral drugs for the treatment of infections with alphaviruses. Several alphaviruses are classified as risk group 3 agents which require handling in high-containment laboratories. To facilitate antiviral screening efforts against alphaviruses, we established a high-throughput antiviral screening assay using reporter Sindbis virus [SINV-GFP; expresses the green fluorescent protein] as a surrogate model for more pathogenic alphaviruses. The assay has strong reproducibility and was validated by reference small-molecule antivirals with various mechanisms of action. The use of high-content imaging as a readout, as demonstrated here, allows for high-throughput antiviral screening and provides a tool for early-stage antiviral discovery against emerging alphavirus threats at a lower biosafety level.

## 1. Introduction

Over the past decades, several human and animal pathogenic arboviruses from different viral families (e.g., dengue virus and Zika virus from *Flaviviridae*; Oropouche virus from *Peribunyaviridae*; and chikungunya virus [CHIKV] from *Togaviridae*) have emerged in new regions, resulting in epidemics. While the emergence of these viruses is multifactorial, climate-related factors are believed to play a significant role [1,2,3].

Rising temperatures, altered precipitation patterns, and ecological disruptions contribute to the proliferation, seasonal persistence, and geographic spread of mosquito vectors [1]. Additional drivers include urbanization, deforestation, increased human and animal mobility, and changes in land use [2,3]. These interconnected factors highlight the importance of a One Health approach integrating human, animal, and environmental health to better understand and mitigate arbovirus emergence and spread.

Among the arboviruses of growing concern are alphaviruses, a genus within the *Togaviridae* family [4]. Alphaviruses are enveloped, positive-sense, single-stranded RNA viruses, many of which are transmitted by mosquitoes and cause disease in a broad spectrum of animals, including humans [4,5]. This genus includes both encephalitic alphaviruses, such as the Venezuelan equine encephalitis virus (VEEV) [6], and arthritogenic alphaviruses, such as CHIKV, the most epidemiologically important alphavirus [5]. CHIKV is responsible for major outbreaks of febrile illness and debilitating polyarthralgia in humans, particularly in tropical and subtropical regions [2,7]. CHIKV infection, while rarely fatal, often leads to chronic arthralgia and long-term disability [8,9,10], reducing quality of life and workforce productivity. The limited availability and applicability of current vaccines against alphaviruses, particularly for children and the elderly who are at greater risk of severe outcomes, highlights the pressing need for antiviral therapeutics.

Surrogate models are safer viral systems that replicate essential features of more pathogenic viruses, enabling antiviral testing under lower biosafety levels. Various surrogate approaches have been used for alphaviruses, including attenuated vaccine strains (e.g., CHIKV 181/25), stable replicon cell lines, and pseudotyped viral particles [11,12,13,14,15]. BSL-2-classified alphaviruses, like Semliki Forest virus (SFV), have also served this purpose [16].

The Sindbis virus (SINV) is a biosafety level 2 (BSL-2)-classified alphavirus. It was first isolated in Egypt in 1952 and it is the prototypical member of the Alphavirus genus within the *Togaviridae* family [4,17,18,19,20,21]. Human SINV infections are typically mild or asymptomatic, occasionally causing fever, rash, and arthralgia [22,23]. Notably, the recent detection of SINV in mosquitoes from southwestern Spain suggests a new introduction of the virus in Europe [24], highlighting its capacity to expand into new regions and underscoring the importance of continued surveillance and research.

SINV serves as a suitable surrogate model for alphavirus drug discovery due to its conserved replication machinery and structural similarity to CHIKV and VEEV [25,26,27,28], allowing initial screening to be conducted safely and efficiently outside of high-containment facilities. We here leverage the use of an SINV strain engineered to express a green fluorescent protein (SINV-GFP) as the basis for a high-content imaging, high-throughput screening (HTS) assay. This approach enables rapid, scalable assessment of antiviral activity while bypassing the constraints associated with BSL-3 pathogens, offering a practical platform for the early-stage identification of candidate antivirals targeting the alphavirus genus.

## 2. Materials and Methods

### 2.1. Cell Lines and Cell Culture

Baby hamster kidney (BHK-21J) cells (generous gift from Peter Bredenbeek, LUMC, The Netherlands; passage number < 20) were cultured in high-glucose DMEM without pyruvate (Gibco, Waltham, MA, USA) supplemented with 10% heat-inactivated fetal bovine serum (FBS; Gibco), 0.75 mg/mL sodium bicarbonate (Gibco), 1 mM sodium pyruvate (Gibco), 25 mM HEPES buffer (Gibco) and 100 U/mL penicillin with 100 µg/mL streptomycin (Thermo Fisher Scientific, Merelbeke-Melle, Belgium).

Human hepatoma 7 (HUH-7) cells (CLS) were cultured in high-glucose DMEM without pyruvate supplemented with 10% heat-inactivated FBS, 0.75 mg/mL sodium bicarbonate, 1 mM sodium pyruvate, and 100 U/mL penicillin with 100 µg/mL streptomycin. The human osteosarcoma (U-2 OS) cells (ATCC, HTB-96) were cultured in McCoy’s 5A Medium Modified (Gibco) supplemented with 10% fetal bovine serum (FBS) and penicillin/streptomycin.

Vero E6 cells (ATCC CRL-1586) were cultured in Minimum Essential Medium (MEM; Gibco) supplemented with 10% heat-inactivated FBS, 0.75 mg/mL sodium bicarbonate, 2 mM L-glutamine (Gibco), 1 mM sodium pyruvate, 1x non-essential amino acids (NEAA; Gibco), and 100 U/mL penicillin with 100 µg/mL streptomycin (Thermo Fisher Scientific).

All cell lines cited above were maintained at 37 °C in a humidified 5% CO_2_ incubator. For all experiments involving viral infection, the respective cell culture media described above were used as the base for infection media supplemented with 2% FBS and with an absence of antibiotics.

### 2.2. Plasmid and Viruses

Recombinant Sindbis virus (strain AR399) expressing GFP was rescued from the pBG218/SINV-GFP plasmid [29], generously provided by Dr. Brian J. Geiss (Colorado State University, Fort Collins, CO, USA). BHK-21J cells were seeded in 6-well tissue culture plates (Falcon) and transfected with pBG218/SINV-GFP using TransIT^®^-LT1 Transfection Reagent (Mirus Bio, Madison, WI, USA) according to the manufacturer’s protocol. Transfected cells were cultured in DMEM supplemented with 2% FBS, without antibiotics, at 37 °C in a 5% CO_2_ incubator. Viral rescue was monitored by GFP expression and the appearance of cytopathic effect (CPE), typically observed 24–48 h post-transfection. These virus preparations were further amplified once in BHK-21J cells to generate working stocks (passage 2), which were used in all subsequent experiments.

The concentration of infectious viral particles for each virus stock was determined using a standard plaque assay on Vero E6 cells. All virus-related procedures were performed under BSL-2 containment, in compliance with national biosafety regulations for pathogen risk classification in Belgium.

### 2.3. Compound Preparation and Dispensing

The following reference compounds were selected based on the literature reports describing antiviral activity against alphaviruses or other RNA viruses and representing distinct mechanisms of action across the viral life cycle: Brequinar (BioSynth, Suzhou, China; a DHODH inhibitor that depletes intracellular pyrimidine pools, impairing viral RNA replication), Pyrimethamine (Sigma-Aldrich, Taufkirchen, Germany; a folic acid antagonist reported to interfere with VEEV capping enzymes and nucleotide biosynthesis, affecting viral RNA synthesis), EIDD-1931 (BLD Pharm, Kaiserslautern, Germany; the active metabolite of Molnupiravir, a nucleoside analog that induces lethal mutagenesis during viral RNA replication), and Hydroxychloroquine (Combi-Blocks, San Diego, CA, USA; a lysosomotropic agent that disrupts viral entry by increasing endosomal pH in vitro). Hydroxychloroquine was dissolved in sterile water, while all other compounds were dissolved in 100% DMSO.

Compounds were spotted into black, clear-bottom, 384-well destination plates (Greiner Bio-One, Kremsmünster, Austria) using the Echo^®^ 650 acoustic liquid handler (Beckman Coulter Life Sciences, Indianapolis, IN, USA), then sealed and stored under desiccation at room temperature. Reference compound plates were initially evaluated using five concentrations (four technical replicates per concentration) to assess assay performance. Based on these preliminary experiments, the setup was subsequently optimized to the eight-concentration format presented in this study.

### 2.4. Cell Susceptibility to SINV-GFP

BHK-21J, HuH-7, U-2 OS, and Vero E6 cells were seeded at a density of 20,000 cells per well in black, clear-bottom, 96-well plates (Greiner Bio-One) in 180 µL of their respective infection media (i.e., culture media supplemented with 2% heat-inactivated FBS and no antibiotics). Cells were incubated overnight at 37 °C in a humidified 5% CO_2_ incubator. The following day, SINV-GFP virus stock was serially diluted 1:10 directly on the plate. At 1 and 2 d.p.i., cells were fixed with 2% formaldehyde for 1 h at room temperature, followed by undergoing a PBS wash. Nuclei were stained with 1 µg/mL 4′,6-diamidino-2-phenylindole (DAPI; Invitrogen, Belgium) for 30 min. Plates were imaged at 10× magnification using the ArrayScan XTI high-content screening system (Thermo Scientific, Belgium).

### 2.5. Optimization of High-Content Imaging-Based Antiviral Assay for SINV-GFP Infection in BHK-21J Cells

BHK-21J cells were seeded in black, clear-bottom, 96-well plates at a density of 20,000 cells per well in 180 µL of infection medium (DMEM-based and supplemented with 2% heat-inactivated FBS and no antibiotics). After overnight incubation at 37 °C in a humidified atmosphere with 5% CO_2_, SINV-GFP was added to the cells via serial 1:10 dilutions, starting from a 1:10 dilution, by adding 20 µL of virus suspension directly to each well, with 6 replicates per virus dilution.

From these 6 replicates, 3 of them were treated with 3 µM of reference antiviral compound EIDD-1931, added simultaneously with viral inoculation. Following virus adsorption and compound exposure, cells were incubated for 2 days at 37 °C in a humidified atmosphere with 5% CO_2_. At 2 d.p.i., cells were fixed with 2% formaldehyde for 1 h at room temperature, washed three times with 1× PBS (pH 7.4), and stained with DAPI (1 µg/mL) for nuclear visualization. Plates were imaged using the ArrayScan XTI high-content screening system (Thermo Fisher Scientific) at 10× magnification, acquiring four fields per well.

Image analysis was performed using the SpotDetector module in HCS Studio software. Nuclei were identified based on DAPI staining (386/23 nm) to define total cell count. GFP-positive cells (488/20 nm) were detected using a fixed mask size of 4, corresponding approximately to nuclear size, and infection was determined by overlapping GFP signal with DAPI-defined nuclei. Cells located at the borders of the fields were excluded from analysis. Infection percentages were calculated by normalizing GFP-positive cells to the total number of DAPI-positive nuclei per well, using infected positive and negative controls to define baseline and maximal infection signals.

### 2.6. One-Step SINV-GFP-Based High-Throughput Screening (HTS) Assay

A BHK-21J cell suspension of 1 × 10^5^ cells/mL was dispensed into the pre-spotted plates at 50 µL per well using a Multidrop™ Combi Reagent Dispenser (Thermo Fisher). For infection, SINV-GFP was diluted to a multiplicity of infection (MOI) of 0.1 and added at 10 µL per well (except for the non-infected control wells which received 10 µL of virus-free medium). Plates were incubated at 37 °C with 5% CO_2_. At 20 h post-infection (h.p.i.), cells were fixed by adding 40 µL per well of 10% formaldehyde in 1× PBS and incubated for 1 h at room temperature under a chemical hood. Plates were washed with a Biotek 405 TS plate washer and stained with 30 µL of DAPI (1 µg/mL in PBS) for 30 min in the dark. Following a final wash, fixed cells were kept in 1× PBS and plates were either imaged immediately or stored at 4 °C for up to one week. Image acquisition and analysis were performed as previously described (Section 2.5).

HCI assay performance was evaluated using the Z′ factor (Z′), a statistical parameter that reflects the separation between the positive control (virus-infected wells without treatment) and the negative control (uninfected wells). Z′ values were calculated for each assay plate based on the infection percentage (GFP-positive cells) using the following formula:

$$Z^{\prime }=1-\;\frac{3\;\times (\sigma_{p}+\sigma_{n})}{\left|{µ}_{p}-{µ}_{n}\right|}$$

where *σ_p_* and *σ_n_* represent the standard deviations of the positive and negative controls, respectively, and *μ_p_* and *μ_n_* are the means. The *Z′* factor between 0.5 and 1 indicates a suitable assay with appropriate separation between controls [30].

For dose–response curve analysis, non-linear regression fitting was performed in GraphPad Prism version 10.0 (GraphPad Software, San Diego, CA, USA). Percent inhibition of infection or cytopathic effect was calculated relative to virus control wells. EC_50_ (half-maximal effective concentration) and CC_50_ (half-maximal cytotoxic concentration) values were derived from a four-parameter logistic (4PL) model with variable slope.

## 3. Results

To identify a suitable cell culture infection model for SINV that is amenable for HTS, several cell lines are assessed for their susceptibility to SINV-GFP infection, which reveal marked differences. BHK-21J cells are most susceptibility to infection, with robust and widespread GFP expression at 2 d.p.i. across viral inocula (Table 1). HuH-7 cells have moderate susceptibility to infection, characterized by a consistent GFP signal and detectable CPE at mid to high virus input (MOI ≥ 0.05). On the other hand, Vero E6 and U-2 OS cells support detectable viral replication only at the two highest MOIs (MOI = 5), indicating limited permissiveness and likely reflecting synchronized, single-cycle infection events. These findings establish BHK-21J as the most permissive cell line to our SINV-GFP and lead to its selection for assay development.

**Figure 1:**
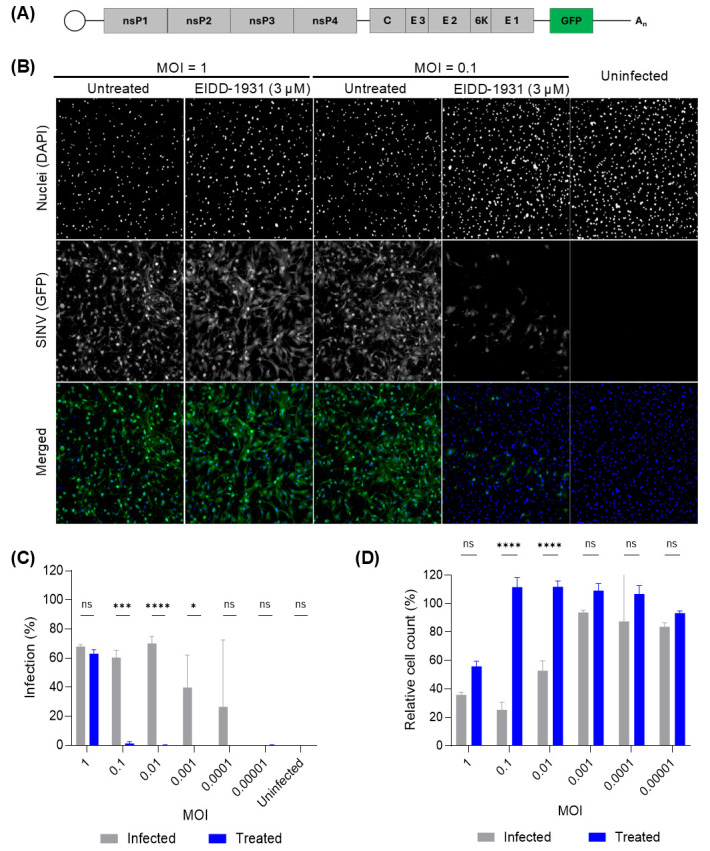
High-content imaging reveals the limitations of single-cycle infection in detecting delayed-acting antivirals. (**A**) Schematic representation of the SINV-GFP reporter genome. The viral RNA is capped at the 5′ end and contains two open reading frames (ORFs). ORF1 encodes the nonstructural polyprotein (nsP1–nsP4), which mediates viral RNA replication. ORF2 encodes the structural polyprotein (C–E3–E2–6K–E1) and is expressed from the subgenomic promoter. In this reporter construct, a duplicated subgenomic promoter drives the expression of the GFP reporter gene. The genome terminates with a 3′ poly(A) tail. (**B**) Representative images of BHK-21J cells infected with SINV-GFP at two different MOIs (1 and 0.1), treated or not treated with EIDD-1931 (3 µM), and acquired 2 d.p.i. Images show DAPI-stained nuclei (top), GFP fluorescence (middle), and merged channels (bottom, Green = SINV, Blue = cell nuclei). While GFP expression remains high and unaffected by EIDD-1931 at MOI = 1, a visible reduction in GFP signal is observed starting from MOI = 0.1. (**C**) Quantification of infection rates (% GFP-positive cells) across viral dilutions in the presence or absence of EIDD-1931, observed two d.p.i. (**D**) Relative cell counts normalized to uninfected control. Cytopathic effects (CPEs) are evident at higher MOIs in untreated wells and are alleviated by EIDD-1931 treatment, consistent with partial protective effects. Bars represent mean ± SD (n = 3). Statistical analysis was performed using two-way ANOVA with Šidák’s multiple comparisons test: * *p* < 0.05; *** *p* < 0.001; **** *p* < 0.0001; ns = not significant.

Following selection of BHK-21J cells for further work, infection parameters were optimized to enable sensitive and reproducible detection of antiviral activity. BHK-21J cells were infected with different virus MOIs and treated with the broader-spectrum inhibitor EIDD-1931. Inhibition of GFP expression was observed only at MOIs ≤ 0.1, while high MOI (MOI = 1) masked antiviral effects (Figure 1A,B). Additionally, untreated cells showed significant CPE at MOIs of 0.1 and 0.01, which were rescued by antiviral treatment (Figure 1C). Together, these results demonstrate that antiviral activity can be effectively assessed using the SINV-GFP-based assay when viral replication occurs over multiple cycles, as is the case at lower MOIs.

To further optimize the assay in a 384-well format, Z′ factors were calculated across different MOIs using 24 replicate wells per condition. This analysis identified an MOI of 0.1 as optimal based on a robust average Z′ value of 0.815 ± 0.035, alongside a favorable infection rate (67.2% ± 1.6%) and moderate cytopathic effect (22.6% ± 7.3%) 20 h.p.i. These parameters confirmed the suitability of MOI 0.1 for all subsequent validation and screening efforts.

**Figure 2:**
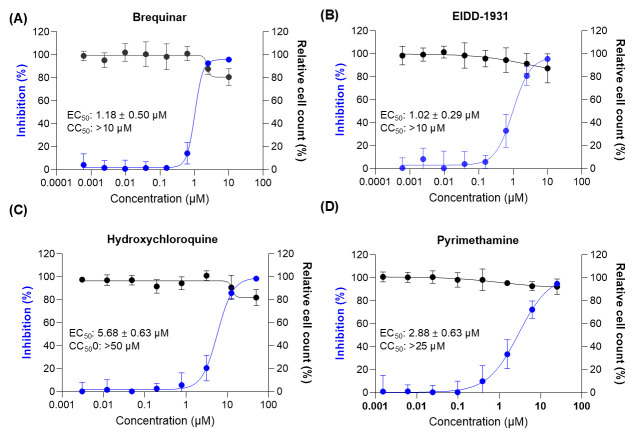
High-content imaging validation of the SINV-GFP assay using reference compounds with previously reported antiviral activity. BHK-21J cells were infected with SINV-GFP (MOI = 0.1) and treated with a five-point dose series of selected compounds. Infection levels were quantified at 20 h post-infection using high-content imaging based on GFP expression. Graphs show percentage inhibition of GFP-positive cells (blue, left axis) and relative cell count normalized to uninfected controls (black, right axis). Data represent mean ± SD of quadruplicate wells. Dose–response curves are shown for: (**A**) Brequinar; (**B**) EIDD-1931; (**C**) Hydroxychloroquine; (**D**) Pyrimethamine. EC_50_ is half-maximal effective concentration and CC_50_ is half-maximal cytotoxic concentration.

To validate the performance of the assay and the dynamic range, four reference compounds (either with direct antiviral activity or targeting the host cell, i.e., EIDD-1931, Brequinar, Hydroxychloroquine [HCQ], and Pyrimethamine) were tested in dose–response format using a dedicated reference plate. The assay consistently achieved a strong infection rate in SINV-GFP-infected controls (71.2 ± 1.5%) with a Z′ factor of 0.70 ± 0.15, confirming robust assay quality. High-content imaging revealed that EIDD-1931 and Brequinar potently inhibited infection, whereas Hydroxychloroquine and Pyrimethamine resulted in weaker effects, mostly at higher concentrations (Figure 2). Quantitative dose–response analysis yielded EC_50_ values of 1.02 ± 0.29 µM for EIDD-1931, 1.18 ± 0.50 µM for Brequinar, 5.60 ± 0.63 µM for HCQ, and 2.88 ± 0.51 µM for Pyrimethamine. All four molecules were relatively well-supported, with cell viability remaining above 75%.

## 4. Discussion

We developed and validated a high-content imaging (HCI) assay based on an SINV-GFP reporter virus to support high-throughput antiviral screening. High-content screening (HCS), combining automated microscopy and image analysis, forms a powerful technology for drug discovery [31]. Unlike conventional bulk-readout assays (e.g., luminescence- or absorbance-based methods), HCI allows for direct visualization and quantification of virus-induced phenotypes, capturing both antiviral activity (via GFP expression as a proxy for SINV replication) and cell viability within the same well [32,33]. Its compatibility with miniaturized formats and automation makes it well-suited for early-stage antiviral discovery.

A key finding during assay development was the important influence of host cell line on assay performance. Despite SINV’s broad tropism [34], we observed marked differences in GFP signal across tested cell lines. This likely reflects not only differential permissiveness to infection but also variability in host-specific factors influencing reporter expression [35,36,37,38]. These findings are consistent with those of Steel et al. (2011) [29], who originally developed the GFP-reporter SINV system and identified BHK cells as the most suitable for virus propagation and GFP visualization.

Another critical variable was the MOI. High-MOI conditions facilitate single-round infection but require large virus stocks and may fail to detect compounds that act over multiple replication cycles. This was evident with EIDD-1931, a nucleoside analog that resulted in minimal antiviral activity at high MOI but with clear antiviral activity at low(er) MOI that required multiple viral replication cycles. These findings emphasize the importance of assay design in ensuring that screening systems are not blind to antiviral mechanisms that require extended replication dynamics.

In BHK-21 cells, SINV infection causes notable cytopathic effects (rounding, detachment, lysis) typically between 10 and 24 h.p.i. [39,40], whereas viral assembly and release begin as early as 4–8 h.p.i [41,42]. To optimize the assay, we aimed to identify conditions that yielded more than 65% infection while keeping the CPE below 30% (cell death was determined by cell count), thereby ensuring a suitable dynamic range for detecting antiviral activity. This was achieved using an MOI of 0.1 and a 20 h endpoint, balancing infection efficiency and minimizing cytopathic effects.

To validate the assay’s capacity to detect diverse antiviral mechanisms, we tested four reference compounds with distinct modes of action: Brequinar (a DHODH inhibitor that depletes nucleotides and modulates immunity [43,44]), Pyrimethamine (a folic acid antagonist reported to inhibit VEEV capping enzymes and adenosine synthesis [45]), EIDD-1931 (the active form of Molnupiravir, a nucleoside analog that induces lethal mutagenesis across various RNA viruses including alphaviruses [46,47,48,49,50,51]), and Hydroxychloroquine (a lysosomotropic agent that disrupts viral entry by raising endosomal pH in vitro [52,53,54,55]). Each compound produced a distinct antiviral profile, demonstrating the assay’s capacity to identify mechanistically diverse inhibitors.

By enabling high-throughput screening under BSL-2 conditions, the SINV-GFP assay facilitates early identification of candidate antivirals that can be prioritized for follow-up in more pathogenic BSL-3 alphaviruses. This tiered approach conserves resources, reduces reliance on high-containment labs, and streamlines the discovery process. Moreover, using a BSL-2 compatible surrogate expands accessibility, helping to democratize antiviral screening by lowering logistical and financial barriers. This approach aligns with key principles of pandemic preparedness, including early threat detection, decentralization of research capacity, and rapid therapeutic development.

## 5. Conclusions

We establish a robust and scalable high-content imaging assay based on an SINV-GFP reporter virus for antiviral screening under BSL-2 conditions. The assay has strong reproducibility and is well-suited for high-throughput applications. Overall, it provides a sensitive and accessible platform for early-stage alphavirus antiviral discovery, offering a valuable starting point for further optimization and downstream development.

## Figures and Tables

**Table 1:** Infection efficiency of SINV-GFP expressed as percentage of GFP-positive cells in different cell lines at varying MOIs and post-infection time points.

	1 d.p.i.				2 d.p.i			
MOI	BHK-21J	HuH-7	U-2 OS	Vero E6	BHK-21J	HuH-7	U-2 OS	Vero E6
5	65.24 ± 1.53	8.10 ± 0.69	7.48 ± 0.47	13.07 ± 0.38	73.39 ± 2.18	24.55 ± 3.98	5.16 ± 2.95	2.26 ± 0.13
0.5	68.32 ± 1.89	2.15 ± 0.35	2.16 ± 0.32	5.15 ± 0.43	77.65 ± 5.41	29.36 ± 2.11	3.03 ± 2.63	2.84 ± 0.71
0.05	62.96 ± 4.99	0.15 ± 0.06	0.33 ± 0.17	0.71 ± 0.34	82.417 ± 0.83	11.74 ± 3.40	0.73 ± 0.30	1.56 ± 0.82
0.005	49.89 ± 8.88	0	0.02 ± 0.01	0.03 ± 0.03	77.05 ± 4.73	3.19 ± 4.37	0.16 ± 0.15	0.15 ± 0.17
0.0005	34.97 ± 8.26	0	0	0	68.17 ± 8.81	0.05 ± 0.09	0.10 ± 0.06	0.02 ± 0.03
0.00005	0	0	0	0	34.93 ± 25.73	0	0	0

Data represent mean ± standard deviation from three replicates. MOI = multiplicity of infection; d.p.i = days post-infection.

## Data Availability

The raw data supporting the conclusions of this article will be made available by the authors on request.
